# Supplementary material for: Modeling glioblastoma heterogeneity as a dynamic network of cell states
Source: Mol Syst Biol. 2021 Sep 16;17(9):e10105. doi: 10.15252/msb.202010105 (PMC8444284; doi:10.15252/msb.202010105)
Supplement: Supplementary file 5 — Source Data for Figure 3 [file MSB-17-e10105-s001.zip › Figure3A_sourcedata/GSEA_3065/nosigdb_state6.GseaPreranked.1622718443993/PARADA_T1_UP_26461091.html]

Details for gene set PARADA\_T1\_UP\_26461091[GSEA]

|  || Dataset | state6 |
| Phenotype | NoPhenotypeAvailable |
| Upregulated in class | na\_neg |
| GeneSet | PARADA\_T1\_UP\_26461091 |
| Enrichment Score (ES) | -0.5942241 |
| Normalized Enrichment Score (NES) | -2.2029884 |
| Nominal p-value | 0.0 |
| FDR q-value | 0.0 |
| FWER p-Value | 0.0 |
Table: GSEA Results Summary

  

Fig 1: Enrichment plot: PARADA\_T1\_UP\_26461091      
 Profile of the Running ES Score & Positions of GeneSet Members on the Rank Ordered List

  

| PROBE | GENE SYMBOL | GENE\_TITLE | RANK IN GENE LIST | RANK METRIC SCORE | RUNNING ES | CORE ENRICHMENT || 1 | TRAF4 |  |  | 244 | 0.163 | -0.0041 | No |
| 2 | NT5DC2 |  |  | 513 | 0.122 | -0.0169 | No |
| 3 | PALLD |  |  | 752 | 0.102 | -0.0292 | No |
| 4 | SLC29A4 |  |  | 780 | 0.100 | -0.0177 | No |
| 5 | TUBB2B |  |  | 795 | 0.099 | -0.0048 | No |
| 6 | CREB5 |  |  | 1231 | 0.074 | -0.0438 | No |
| 7 | CCND1 |  |  | 1317 | 0.071 | -0.0432 | No |
| 8 | IFITM3 |  |  | 1398 | 0.068 | -0.0424 | No |
| 9 | CDK4 |  |  | 1490 | 0.064 | -0.0434 | No |
| 10 | CENPA |  |  | 2199 | 0.042 | -0.1184 | No |
| 11 | MCM6 |  |  | 2484 | 0.034 | -0.1460 | No |
| 12 | S100A11 |  |  | 2523 | 0.033 | -0.1455 | No |
| 13 | TGFBI |  |  | 2809 | 0.026 | -0.1743 | No |
| 14 | VIM |  |  | 2831 | 0.026 | -0.1729 | No |
| 15 | EIF4EBP1 |  |  | 2837 | 0.026 | -0.1697 | No |
| 16 | ARPC1B |  |  | 3027 | 0.021 | -0.1882 | No |
| 17 | CXCL16 |  |  | 3974 | 0.001 | -0.2964 | No |
| 18 | SOCS3 |  |  | 3978 | 0.001 | -0.2965 | No |
| 19 | EN2 |  |  | 3998 | 0.001 | -0.2986 | No |
| 20 | TUBB6 |  |  | 4313 | -0.005 | -0.3338 | No |
| 21 | RHOJ |  |  | 4595 | -0.011 | -0.3644 | No |
| 22 | GTSE1 |  |  | 5195 | -0.022 | -0.4298 | No |
| 23 | LMNB1 |  |  | 5545 | -0.029 | -0.4654 | No |
| 24 | PLEKHG2 |  |  | 5823 | -0.035 | -0.4920 | No |
| 25 | F2R |  |  | 5881 | -0.036 | -0.4933 | No |
| 26 | CDCA2 |  |  | 6175 | -0.042 | -0.5207 | No |
| 27 | SAMD9L |  |  | 6705 | -0.056 | -0.5731 | No |
| 28 | TROAP |  |  | 6707 | -0.056 | -0.5650 | No |
| 29 | BUB1B |  |  | 6717 | -0.056 | -0.5578 | No |
| 30 | PRC1 |  |  | 6918 | -0.062 | -0.5715 | No |
| 31 | PSMB8 |  |  | 6936 | -0.063 | -0.5643 | No |
| 32 | HIST2H2AC |  |  | 6963 | -0.064 | -0.5580 | No |
| 33 | BIRC5 |  |  | 7024 | -0.066 | -0.5552 | No |
| 34 | COL16A1 |  |  | 7352 | -0.076 | -0.5815 | No |
| 35 | NCAPH |  |  | 7438 | -0.079 | -0.5797 | No |
| 36 | LGALS3 |  |  | 7566 | -0.084 | -0.5819 | Yes |
| 37 | USP18 |  |  | 7577 | -0.085 | -0.5706 | Yes |
| 38 | CDCA3 |  |  | 7631 | -0.087 | -0.5639 | Yes |
| 39 | AURKA |  |  | 7700 | -0.091 | -0.5584 | Yes |
| 40 | RNF213 |  |  | 7812 | -0.096 | -0.5570 | Yes |
| 41 | PBK |  |  | 7905 | -0.101 | -0.5527 | Yes |
| 42 | CCNB1 |  |  | 7958 | -0.104 | -0.5433 | Yes |
| 43 | CDK2 |  |  | 8018 | -0.108 | -0.5343 | Yes |
| 44 | PARP14 |  |  | 8063 | -0.111 | -0.5231 | Yes |
| 45 | MCM2 |  |  | 8099 | -0.114 | -0.5104 | Yes |
| 46 | KIF2C |  |  | 8328 | -0.135 | -0.5167 | Yes |
| 47 | CKAP2 |  |  | 8330 | -0.136 | -0.4970 | Yes |
| 48 | HMGB2 |  |  | 8388 | -0.145 | -0.4823 | Yes |
| 49 | TAGLN2 |  |  | 8457 | -0.153 | -0.4676 | Yes |
| 50 | KIF23 |  |  | 8465 | -0.154 | -0.4458 | Yes |
| 51 | GPX7 |  |  | 8596 | -0.189 | -0.4331 | Yes |
| 52 | IGFBP3 |  |  | 8615 | -0.197 | -0.4063 | Yes |
| 53 | EMP1 |  |  | 8641 | -0.209 | -0.3786 | Yes |
| 54 | NES |  |  | 8677 | -0.227 | -0.3495 | Yes |
| 55 | IGFBP2 |  |  | 8719 | -0.260 | -0.3161 | Yes |
| 56 | ANXA2 |  |  | 8741 | -0.279 | -0.2777 | Yes |
| 57 | LGALS3BP |  |  | 8744 | -0.282 | -0.2367 | Yes |
| 58 | TIMP1 |  |  | 8751 | -0.290 | -0.1950 | Yes |
| 59 | CD9 |  |  | 8762 | -0.303 | -0.1518 | Yes |
| 60 | CD63 |  |  | 8776 | -0.335 | -0.1043 | Yes |
| 61 | SPP1 |  |  | 8779 | -0.338 | -0.0551 | Yes |
| 62 | TNC |  |  | 8787 | -0.389 | 0.0010 | Yes |
Table: GSEA details [plain text format]

  

Fig 2: PARADA\_T1\_UP\_26461091: Random ES distribution      
 Gene set null distribution of ES for **PARADA\_T1\_UP\_26461091**
